# ABCB1 inhibition provides a novel therapeutic target to block TWIST1-induced migration in medulloblastoma

**DOI:** 10.1093/noajnl/vdab030

**Published:** 2021-04-28

**Authors:** Aishah Nasir, Alice Cardall, Ramadhan T Othman, Niovi Nicolaou, Anbarasu Lourdusamy, Franziska Linke, David Onion, Marina Ryzhova, Hanna Cameron, Cara Valente, Alison Ritchie, Andrey Korshunov, Stefan M Pfister, Anna M Grabowska, Ian D Kerr, Beth Coyle

**Affiliations:** 1 Children’s Brain Tumour Research Centre, Division of Child Health, Obstetrics and Gynaecology, School of Medicine, University of Nottingham, Nottingham, UK; 7 College of Medicine, University of Duhok, Kurdistan, Iraq; 2 Division of Cancer and Stem Cells, School of Medicine, University of Nottingham, Nottingham, UK; 3 School of Life Sciences, University of Nottingham, Nottingham, UK; 4 Department of Neuropathology, NN Burdenko Neurosurgical Institute, Moscow, Russia; 5 Cooperation Unit Neuro-oncology, German Cancer Research Center (DKFZ), Heidelberg, Germany; 6 Hopp Children’s Cancer Center Heidelberg (KiTZ), German Cancer Research Center (DKFZ), Division of Pediatric Neurooncology and Heidelberg University Hospital, Department of Pediatric Hematology and Oncology, Heidelberg, Germany

**Keywords:** ABCB1, epithelial–mesenchymal transition, Harmine, medulloblastoma, TWIST1, 3D-BME model

## Abstract

**Background:**

Therapeutic intervention in metastatic medulloblastoma is dependent on elucidating the underlying metastatic mechanism. We investigated whether an epithelial–mesenchymal transition (EMT)-like pathway could drive medulloblastoma metastasis.

**Methods:**

A 3D Basement Membrane Extract (3D-BME) model was used to investigate medulloblastoma cell migration. Cell line growth was quantified with AlamarBlue metabolic assays and the morphology assessed by time-lapse imaging. Gene expression was analyzed by qRT-PCR and protein expression by immunohistochemistry of patient tissue microarrays and mouse orthotopic xenografts. Chromatin immunoprecipitation was used to determine whether the EMT transcription factor TWIST1 bound to the promoter of the multidrug pump *ABCB1*. TWIST1 was overexpressed in MED6 cells by lentiviral transduction (MED6-TWIST1). Inhibition of ABCB1 was mediated by vardenafil, and TWIST1 expression was reduced by either Harmine or shRNA.

**Results:**

Metastatic cells migrated to form large metabolically active aggregates, whereas non-tumorigenic/non-metastatic cells formed small aggregates with decreasing metabolic activity. TWIST1 expression was upregulated in the 3D-BME model. TWIST1 and ABCB1 were significantly associated with metastasis in patients (*P* = .041 and *P* = .04, respectively). High nuclear TWIST1 expression was observed in the invasive edge of the MED1 orthotopic model, and TWIST1 knockdown in cell lines was associated with reduced cell migration (*P* < .05). TWIST1 bound to the *ABCB1* promoter (*P* = .03) and induced cell aggregation in metastatic and TWIST1-overexpressing, non-metastatic (MED6-TWIST1) cells, which was significantly attenuated by vardenafil (*P* < .05).

**Conclusions:**

In this study, we identified a TWIST1–ABCB1 signaling axis during medulloblastoma migration, which can be therapeutically targeted with the clinically approved ABCB1 inhibitor, vardenafil.

**Key Points**

- TWIST1 and ABCB1 are significantly associated with medulloblastoma metastasis.
- Overexpression of TWIST1 in non-metastatic cells increased cell aggregation.
- Inhibiting ABCB1 with vardenafil in a 3D-BME model attenuated cell aggregation.

**Importance of the Study**

Here, we present a 3D culture system that uses tumor-derived basement membrane extract (BME) to provide a representative in vitro microenvironment for medulloblastoma growth and metastasis studies reducing the reliance on in vivo studies. We show that this 3D-BME model can be used to accurately distinguish between non-tumorigenic/non-metastatic and metastatic cell lines based on morphological and transcriptional changes. We provide the first conclusive evidence that the EMT factor, TWIST1 plays a key regulatory role during medulloblastoma metastasis, supporting the existence of a migratory EMT-like pathway. Furthermore, by identifying a direct link between the transcription factor TWIST1 and the multidrug transporter ABCB1, we provide novel therapeutic targets which can be exploited by re-purposing the ABCB1-specific inhibitor vardenafil to block medulloblastoma cell migration. These findings provide evidence for a novel therapeutic strategy that could be applied to the clinic for treating metastatic chemo-incurable medulloblastoma tumors.

Medulloblastomas are aggressive malignant pediatric brain tumors of the cerebellum that have a high propensity to metastasize (one-third of newly diagnosed medulloblastoma patients present with metastatic disease).^1,2^ Patient response to current multimodal therapies which combine surgery, chemotherapy, and craniospinal radiotherapy is extremely poor, while long-term survivors are left with debilitating side effects (eg, neurocognitive deficits) or have a high likelihood of developing recurrent tumors. There is an urgent need to improve patient outcomes and eliminate treatment-related neurotoxicity by identifying therapies that can target specific molecular mediators of metastasis.

Molecularly distinct subgroups are associated with a differential propensity to metastasize and are paramount in the determination of high-risk tumors.^3^ Leptomeningeal dissemination is a well-recognized route of metastasis whereby tumor cells disseminate to the leptomeninges of the brain and spinal cord via the cerebrospinal fluid.^4^ Emerging evidence has also identified a vascular-mediated route of dissemination whereby circulating medulloblastoma cells from the primary tumor were present in the bloodstream as observed in the invasion–metastasis cascade, a metastatic process characteristic of epithelial tumors.^5,6^ This suggests that pathways of metastasis identified in epithelial tumors including the Epithelial–Mesenchymal Transition (EMT) pathway, a migratory process that initiates the invasion–metastasis cascade,^7^ could also be relevant during metastasis of neuroectodermal medulloblastomas.

During EMT, cells reversibly switch from adhesive epithelial to motile mesenchymal derivatives. In other nonepithelial tumors including glioblastoma,^8^ activation of EMT markers promotes mesenchymal change in an EMT-like process that regulates stemness, invasion, and therapy resistance.^9^ Identification of several EMT-associated markers including BMI1,^10^ FOXG1,^11^ MMP9,^12,13^ Rac1/PAK1,^14^ and the uPA/uPAR^15^ complex in medulloblastomas also supports activation of this process.

Three-dimensional (3D) culture systems provide a more biologically relevant model compared to cells in 2D culture; signals from an extracellular matrix (ECM) can restore tissue-specific gene expression and function since cell–cell and cell–matrix interactions are more representative of the original tumor microenvironment.^16,17^ Here we applied a 3D preclinical tumor model originally described by Sasser et al.,^18^ where tumor cells are embedded within low-stiffness, laminin-rich ECM that incorporates key components present in the basement membrane of blood vessels and at the leptomeninges (sites of medulloblastoma metastasis).^19^ Using this model, initiation of an EMT-like process was investigated across a panel of medulloblastoma cell lines derived from both non-metastatic and metastatic (taken from the primary tumor or metastases) patient tumor tissue and non-tumorigenic cerebellar progenitors.

We found that only metastatic cell lines migrated to form viable 3D aggregates and during this process, the early-stage EMT transcription factor TWIST1 was upregulated. Expression of TWIST1 and the ATP-binding cassette sub-family B member 1 (ABCB1) multidrug pump (a putative TWIST1 downstream target) correlated with poor clinical outcome. We confirmed by chromatin immunoprecipitation (ChIP) analysis that TWIST1 binds to the promoter region of ABCB1 and furthermore showed that TWIST1-induced cell migration could be blocked by knocking down TWIST1 and by targeting TWIST1 or ABCB1 with Harmine or the FDA-approved phosphodiesterase-5 inhibitor vardenafil, respectively. Our work provides evidence that ABCB1 inhibition could provide a promising therapeutic strategy to target TWIST1-induced metastasis in medulloblastoma.

## Materials and Methods

### Patient Characteristics

Clinical and histological data for primary tumor patient samples for the Birmingham cohort are outlined in Supplementary Table S1. Clinical details of patients included in tissue microarrays (TMAs) obtained from the Children’s Brain Tumour Research Centre (CBTRC), Nottingham and German Cancer Research Centre, DKFZ were previously published by Othman et al.^20^ and Dubuc et al.,^21^ respectively. These studies, and the experimental protocols required, were reviewed and approved by the National Research Ethics Service Committee East Midlands—Nottingham 2 and the Ethics Committee of the Burdenko Neurosurgical Institute (Ethical vote number 563/6–16) and those of the University of Heidelberg, in compliance with the Russian Federation and German regulations of Health Insurance Portability. Both studies were in accordance with the ethical standards laid down in an appropriate version of the 1975 Declaration of Helsinki, as revised in 1983. For all patients, informed consent was obtained from the patient, or a parent and/or legal guardian where the patient was younger than 18 years of age, prior to their inclusion in the study.

### Cell Culture

FB83 from human fetal brain tissue and MED1 and MED6 cell lines from patient medulloblastomas were derived at the CBTRC as previously described^22,23^ with approval from Local Research Ethics Committee and cultured in tumor media Dulbecco’s modified Eagle’s medium (DMEM, GIBCO: 11885084) containing 15% (v/v) fetal bovine serum (FBS, Hyclone). MED6 cells were stably transduced in-house with a TWIST1 expression vector (based on the pLVX-IRES-tdTomato plasmid construct from Clontech) according to the manufacturer’s instruction to generate the MED6-TWIST1 cell line which was cultured in tumor media. UW228-3 was provided by Dr John R. Silber and cultured as recommended.^24^ The C17.2 cell line generated from mouse cerebellar progenitor cells was provided by Dr Evan Snyder (Harvard Medical School, Massachusetts) and cultured as recommended.^25,26^ D283Med^27^ (ATCC) and D458Med^28^ were provided by Dr Darell Bigner, Duke University and maintained in DMEM supplemented with 10% FBS. ONS-76 were provided by Dr Annette Künkele, Charité Universitätsmedizin Berlin, Germany and maintained in RPMI-1640 medium (Sigma-Aldrich: R8758), supplemented with 10% FBS. D283Med and ONS-76 cell lines were transduced with TWIST1-shRNA GIPZ Lentiviral clone (V3LHS_329863; Dharmacon). About 48 h after infection, the cells were selected using 1 µg/ml of puromycin and the knockdown was confirmed by PCR and western blot analysis.

### Murine Metastatic Medulloblastoma Model

The murine model was produced using MED1-Fluc cells generated by lentiviral transduction of MED1 cells with the in-house luciferase expression vector (pLVX-luc) based on the Lenti-X^TM^ Lentiviral Expression System (Clontech) according to manufacturer’s instructions to allow visualization in vivo. Full experimental methods are detailed in Supplementary Methods in accordance with the ARRIVE guidelines. MED1-Fluc cells with the viability of more than 90%, which had been maintained in vitro in tumor media and selected with puromycin (4 µg/ml) weekly, were injected at 6 × 10^4^ cells in 5 µl of PBS (12 × 10^6^ cells/ml) intracranially (at 7 mm posterior to bregma, 1 mm right of the midline, 3 mm deep using a 26-gauge Hamilton Gastight 1701 syringe needle) into each MF-1/CD-1 nude male mouse (obtained from Harlan, UK at 8–12 weeks of age). Tumor growth was monitored with bioluminescence whole-body imaging of mice, performed twice weekly upon d-Luciferin (120 mg/kg by weight) injection using the IVIS Spectrum (Caliper Life Sciences). Mice were monitored daily and euthanized upon showing any signs of adverse effects (eg, neurological deficits). All animal experiments were performed in accordance with the United Kingdom Animals (Scientific Procedures) Act 1986, under the UK Home Office project license authority PPL40/3559. Ethical approval was granted by the University of Nottingham Animal Welfare and Ethical Review Board.

### Immunohistochemistry

Sections of patient brain tumor tissue, TMAs, murine brain, and spinal column (10% decalcification) containing MED1-Fluc xenografts were stained with anti-TWIST1 (ABD29; Millipore; 1:250–1:500). Nottingham and German TMAs were stained with anti-ABCB1 (C219, Calbiochem; 1:40; Supplementary Table S2). All immunohistochemistry experiments were conducted using the Dako Envision Detection kit (DAKO REAL EnVision) as previously described by Othman et al.^20^

### Immunofluorescence

MED6 and MED6-TWIST1 cells plated in 8-well chamber slides (6 × 10^3^ cells/well) were fixed in 4% paraformaldehyde (Sigma) for 15 min, permeabilized with 0.1% v/v Triton-X100 in PBS (PBS-T; Sigma) for 10 min, and blocked with 2% v/v normal goat serum (Life Technologies) in PBS-T for 1 h. The primary antibody anti-TWIST1 (1:250) was added to cells for 1 h, rinsed with PBS, and subsequently incubated with Alexa Fluor 488 anti-rabbit (1:400; Life Technologies) for 1 h. Cells were then mounted using Vectashield with 4′,6-diamidino-2-phenyl-indole (Vector Laboratories) and visualized with inverted fluorescence microscopy (Nikon Eclipse Ti-U).

### 3D-BME Migration Assay

Cells were resuspended in ice-cold Cultrex basement membrane extract (BME; 3 mg/ml; Trevigen) diluted in RPMI-1640 supplemented with 1% w/v l-glutamine and plated (100 µl/well) at appropriate densities (Supplementary Table S3) in low-adherent, black-walled, clear-bottom, 96-well plates (BrandTech 781671) pre-warmed to 37°C. Plates were incubated (5% CO_2_ at 37°C) for 30 min to facilitate BME polymerization before 50 µl of drugs (CCT007093 [Axon Med Chem], Vardenafil [LKT Laboratories]) diluted in RPMI-1640 supplemented with 1% l-glutamine were added for 96 h exposure. The AlamarBlue assay (Invitrogen; 10% v/v, 37°C for 1 h) was used to assess metabolic activity daily using a fluorescent plate reader (excitation 560 nm, emission 588 nm, Flex Station II, Molecular Devices).

### 3D Transwell Migration Assay

D283Med and ONS-76 cells were pretreated with 5 µM or 20 µM of Harmine (Abcam) respectively and incubated (5% CO_2_ at 37°C) for 48 h. Cells were re-treated with Harmine in a medium containing 2% FBS and incubated for a further 24 h. Transwell inserts (Greiner; 8 µm) were coated with Collagen IV (Fisher; 0.2 µg/insert) and Laminin (AMS Bio 5 µg/ insert) in a 24-well plate (Greiner). 1 × 10^5^ viable cells were plated on the inserts with the outer chamber filled with medium containing 10% FBS and cells were allowed to migrate for 48 h. Media were removed from the chambers and migrated cells dislodged from the bottom surface of the insert by adding 1× cell dissociation solution (R&D Systems; 5% CO_2_ at 37°C for 1 h). The PrestoBlue assay (Invitrogen; 10% v/v, 37°C for 10 min) was used to assess metabolic activity and quantify cell migration using a fluorescent plate reader (excitation 544 nm, emission 590 nm).

### Phase Microscopy and Image Analysis

3D-time-lapse images were captured daily using a Nikon Eclipse Ti inverted microscope. Images acquired for quantification consisted of 3 separate fields obtained for each well (*n* = 3) from 3 independent experiments. XYZ coordinates were fixed on the initial day of set up (day 0) and used to create composite images (z-stacks). Cell growth was analyzed by converting images into black and white, thresholding using the Yen algorithm^29^ and quantifying the mean aggregate area per condition using the open-source Fiji software (http://fiji.sc/Fiji). Time-lapse videos were taken between days 1 and 6, by automatically capturing images per well at 70- to 100-min intervals, to assess cell aggregation (Cell-IQ CM Technologies Oy).

### Quantitative Real-Time Polymerase Chain Reaction

RNA isolation of cell lines with high yield under standard conditions was performed using the mirVana miRNA isolation kit (Ambion). RNA isolation of cells with low yield cultured in the 3D-BME model was lysed with TRI-reagent (Sigma-Aldrich; 1 ml per 5–10 × 10^6^ cells) and chloroform, precipitated with isopropanol and 70% ethanol washes and re-suspended in nuclease-free water. RNA samples were transcribed into cDNA using reverse transcriptase (Superscript III; Invitrogen). Gene expression of the resultant cDNA template was assessed by quantitative reverse transcription PCR (CFX96 real-time PCR machine; BIORAD) and iQ SYBR SuperMix (BIORAD). Primer sequences are summarized in Supplementary Table S4. The house-keeping gene GAPDH was used as a control to normalize the data and the relative mRNA expression level was calculated using the ΔCt method.

### Chromatin Immunoprecipitation

D283Med cells were crosslinked with 0.75% paraformaldehyde for 7 min, lysed, and sonicated to obtain 200–500 bp chromatin fragments. Protein G beads (ThermoFisher Scientific) were incubated overnight at 4°C with either 5 μg of anti-TWIST1 (TWIST2C1α) antibody (Abcam), negative control IgG1 antibody (Cell Signalling Technology), or H3KMe4 antibody (Active Motif), before combining with 25 μg of chromatin fragments and incubating overnight at 4°C. The antibody–protein–DNA complexes were eluted from the beads and crosslinks reversed using 0.3M NaCl at 65°C. DNA was purified by phenol–chloroform extraction and subjected to qRT-PCR using primers (Supplementary Table S5) specific to E-box binding sites within the promoter regions of *ABCB1*, *GAPDH*, and *SNAI2*. Active promoter regions were confirmed using the H3K4Me3 antibody.

### Statistical Analysis

Patient TMA data were analyzed in SPSS version 24 statistical software (IBM). ABCB1 and TWIST1 expression were compared against metastatic status (M0/M+) using Pearson’s chi-squared test. All remaining statistical differences were calculated using GraphPad Prism version 7.0. Results were presented as mean ± standard error. Statistical differences for the relative aggregation rate between conditions at each time point were assessed using unpaired *t*-tests corrected for multiple comparisons using the Sidak–Bonferroni method. ChIP was assessed using a Student’s paired *t*-test. Gene expression comparison between groups was assessed using one-way analysis of variance with Tukey’s multiple comparisons post-hoc test. *P* values ≤.05 were considered significant.

## Results

### Metastatic Medulloblastoma Cells Form Metabolically Active Aggregates in a 3D-BME Model

**Figure 1.**
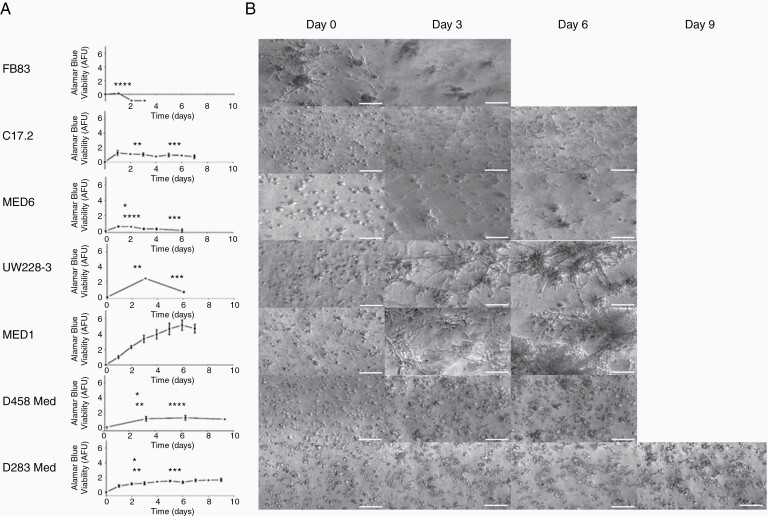
Cell growth and morphology in the 3D-BME model. Non-tumorigenic (FB83 and C17.2), non-metastatic (MED6 and UW228-3), and metastatic (MED1, D458Med, and D283Med) cell lines were cultured in the 3D-BME model. (A) Metabolic activity was assessed with the AlamarBlue metabolic assay to determine the arbitrary fluorescent units (AFU; *n* ≥ 2). One-way ANOVA statistical tests shown were compared with MED1 (denoted in black), FB83 (denoted in red), and UW228-3 (denoted in blue) at days 3 and 6 (*P ≤ .05, **P ≤ .001, ***P ≤ .0005, and ****P ≤ .0001). (B) Morphology with time-lapse images taken at ×10 magnification (scale bars represent 100 µm).

Culturing cells in 3D tumor microenvironment models has been shown to better represent cell growth, morphology, and transcriptional profiles of the tumor. In this study, we investigated whether previously defined non-tumorigenic (C17.2^25^ and FB83^23^), non-metastatic (MED6^20^ and UW228-3^24^), and metastatic (MED1^20^, D283Med^27^ and D458Med^28^) cell lines could be distinguished based on the aforementioned factors. The AlamarBlue assay and time-lapse imaging were used to assess metabolic activity (Figure 1A) and morphology (Figure 1B), respectively, of each cell line cultured in serum-free 3D-BME. Metastatic cell lines (MED1, D458Med, and D283Med) formed metabolically active cell aggregates viable for 6–9 days, while non-tumorigenic/non-metastatic cell lines either formed cell aggregates viable for only 3 days (FB83, MED6, and UW228-3) or remained as single cells (C17.2). Live-cell imaging (CELL-IQ) of the metastatic MED1 cell line, which formed large metabolically active aggregates, captured cell interactions formed during 6 days of culture in the 3D-BME model. During the early days of culture (days 1–3; Supplementary Video S1), cell aggregates increased in size by sequestering single cells to the aggregate body. Later days of culture (days 3–6; Supplementary Video S2) promoted aggregates to rapidly increase in size by combining, corroborating with reported metastatic phenotypes.^30^

### WIP1 Inhibition Blocks Aggregation in the 3D-BME Model

**Figure 2.**
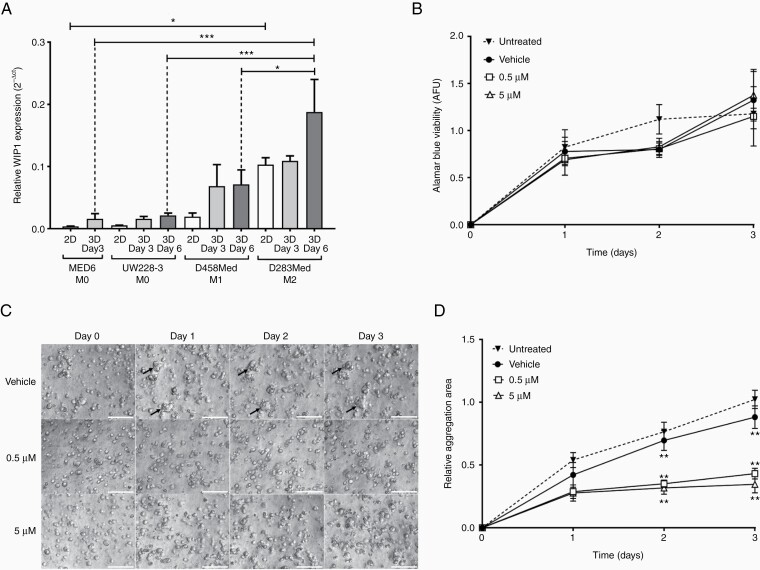
Investigating the metastatic marker *WIP1* in the 3D-BME model. (A) Relative gene expression of *WIP1* was assessed in 2D and 3D samples of medulloblastoma cell lines normalized against *GAPDH* using the 2^−ΔCt^ method (one-way ANOVA analysis with Tukey’s multiple comparisons post-hoc test; *P ≤ .05 and ***P ≤ .001; *n* = 3). (B–D) D283Med were exposed to CCT007093 (0.5 µM and 5 µM), vehicle (0.1% DMSO), or untreated at days 0–3 in the 3D-BME model. (B) Metabolic activity was assessed with the AlamarBlue metabolic assay for each condition (*n* = 3). (C) Time-lapse images of vehicle (black arrows denote cell aggregates) and treated D283Med cells at ×10 magnification (scale bars represent 100 µm). (D) The mean aggregate area of D283Med was quantified relative to day 0 (using the open-source FIJI software) from time-lapse images (*n* = 3).

Overexpression of the EMT-associated marker WIP1 (wild-type p53-induced protein1) promotes cell migration and invasion in metastatic medulloblastoma patients and 2D cell migration models.^31,32^ To validate the 3D-BME model of medulloblastoma metastasis, we assessed whether expression of WIP1 increased in metastatic cell lines grown in 3D-BME and if the cells’ aggregation phenotype could be blocked by inhibiting WIP1. In 2D culture, WIP1 levels were highest in D283Med cells. In the 3D-BME model, WIP1 levels increased across all cell lines compared to 2D culture, and the metastatic cell lines (D283Med and D458Med) showed increased WIP1 levels compared to the M0 cell lines (Figure 2A).

To block cell aggregation in the 3D-BME model, the high WIP1-expressing D283Med cell line was treated with a WIP1-specific small molecule inhibitor, CCT007093 for 72 h^32^ at nontoxic concentrations (Figure 2B). Exposure to CCT007093 significantly attenuated D283Med cell aggregation at days 2 and 3 (Figure 2C and D; *P* ≤ .01) compared to vehicle-treated cells, validating the 3D-BME model as modeling aspects of medulloblastoma metastasis.

### The EMT Transcription Factor TWIST1 Is a Marker of Cell Aggregation and a Driver of Medulloblastoma Migration

Activation of the EMT migratory program has been associated with metastasis of several cancers including medulloblastoma, leading us to investigate the expression of transcription factors (*SNAI2* and *TWIST1*) that are expected to initiate the EMT program.^33^ The publicly available Pfister^34^ and Roth^35^ patient datasets revealed lower *SNAI2* expression in medulloblastoma patient samples compared to the normal cerebellum, whereas *TWIST1* expression was significantly increased in medulloblastoma samples. *SNAI2* expression was not observed below the detection limit in 2D or 3D samples of the C17.2 or MED1 cell lines. High expression of TWIST1 was, however, observed in the metastatic MED1 cell line and increased significantly in the 3D-BME culture (Supplementary Figure S1).

**Figure 3.**
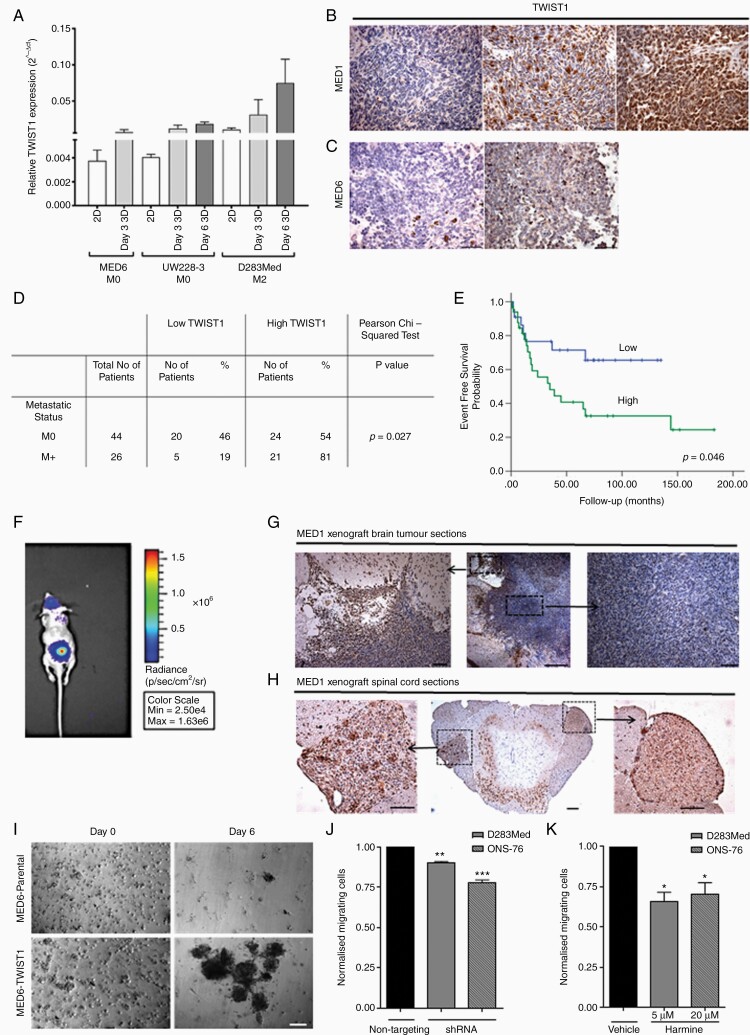
TWIST1 expression and functional analysis. (A) Relative gene expression of *TWIST1* was assessed in 2D and 3D samples of 3 medulloblastoma cell lines and normalized against *GAPDH* using the 2^−ΔCt^ method. (B) Representative images of nuclear TWIST1 IHC staining of MED1 and (C) MED6 patient tumor tissue at ×20 magnification (scale bars represent 50 µm). (D) Nuclear TWIST1 IHC staining of Nottingham and Birmingham TMA’s was scored and correlated with metastatic status (M0/M+) in 70 patients (>3 years old; Pearson’s chi-squared exact test; *P =* .027). (E) High TWIST1 expression correlated to adverse EFS in Kaplan–Meier curves of Nottingham and Birmingham TMA’s (Log-rank test; *P* = .046). (F) Bioluminescent images (obtained at 60 s exposure time) of brain and spinal cord tumors at 21 days after implanting MED1-Fluc cells into the mouse cerebellum. (G and H) Nuclear TWIST1 IHC staining of horizontal sections of the (G) brain and (H) right dorsal ganglia tumor of the spinal cord taken at ×4 and ×20 magnification (scale bars shown represent 50 µm). (I) MED6 and MED6-TWIST1 morphology with time-lapse images taken at ×10 magnification (scale bars represent 100 µm). (J and K) TWIST1 was inhibited in D283Med and ONS-76 cell lines using (J) shRNA or (K) Harmine (D283Med: 5 µM and ONS-76: 20 µM) for 72 h, before plating (1 × 10^5^ cells/insert) onto a Collagen IV and Laminin coated transwell insert. Migrating cells were compared to either a non-targeting shRNA or DMSO control (paired Student’s *t*-test *P ≤ .05, **P ≤ .01; *n* ≥ 2).

TWIST1 gene expression was assessed in 2D and 3D samples of a further 3 cell lines (MED6, UW228-3, and D283Med; Figure 3A). In the metastatic cell line (D283Med), TWIST1 gene expression was upregulated during 3D-BME culture. TWIST1 protein expression was also assessed in primary tumor samples corresponding to the MED1 and MED6 cell lines. High nuclear expression of the TWIST1 transcription factor was observed only in the MED1 primary tumor (Figure 3B and C).

TWIST1 protein expression was then assessed in a TMA of 70 medulloblastoma patients from Nottingham and Birmingham hospitals. Of 70, 45 (64%) samples showed high expression (>30% nuclear positivity) of TWIST1. About 81% of patients with metastatic disease (M+) showed high TWIST1 expression while only 54% of patients categorized as M0 at the time the primary tumor was resected showed high TWIST1 expression. A chi-squared test and Kaplan–Meier analysis showed that TWIST1 expression significantly correlated with metastasis and worse event-free survival (EFS), respectively (*P* = .027 and .046, respectively; Figure 3D and E). Metastatic disease and molecular subgroup were also significantly correlated with EFS and overall survival (Supplementary Table S6). Follow-up information on 36 of the 44 patients categorized as M0 revealed that only 23% of patients who showed low TWIST1 expression developed metastatic disease, whereas 58% of patients with high TWIST1 expression later developed metastatic disease (*P* = .037; Supplementary Figure S2) further highlighting the possible importance of TWIST1 expression as a prognostic biomarker in medulloblastoma patients.

TWIST1 protein expression was also assessed in xenograft tissue taken from an in vivo orthotopic model of MED1 which developed spinal cord metastases (Figure 3F–H). TWIST1 expression was observed at the invasive edge (Figure 3G) but not in the core (Figure 3G and G) of the primary xenograft and in the dorsal root ganglia of spinal tumors (Figure 3H and H).

**Figure 4.**
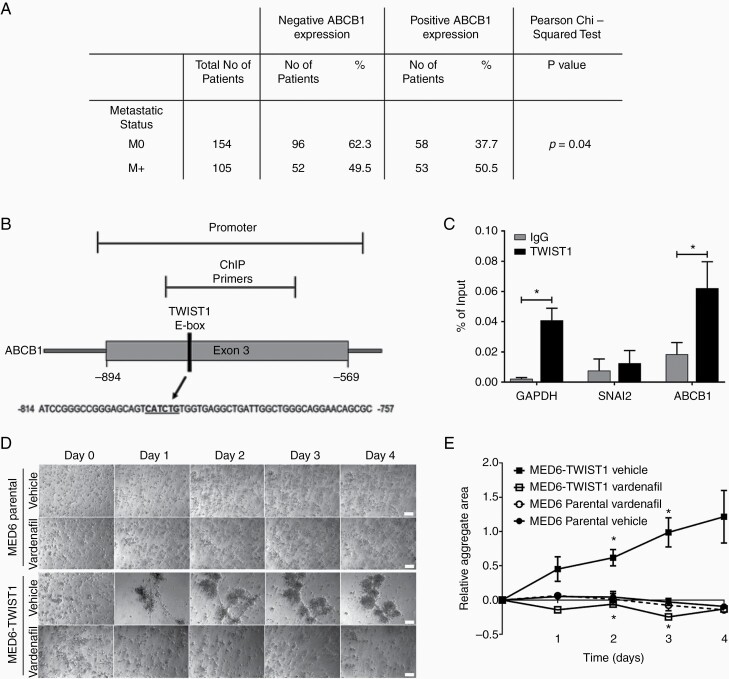
TWIST1 regulates *ABCB1*. (A) Membranous ABCB1 IHC staining of DKFZ TMA and Nottingham TMA was scored and correlated with metastatic status (M0/M+) in a total of 259 patients (Pearson’s chi-squared exact test *P* = .04). (B) Schematic of the E-box binding site in the Exon 3 promoter region of *ABCB1.* (C) ChIP qPCR analysis of the promoter regions in *GAPDH*, *SNAI2*, and *ABCB1*, showing enrichment with TWIST1 antibody for *GAPDH* and *ABCB1* compared with IgG antibody control. Data are expressed as a percentage of the input (paired Student’s *t*-test *P ≤ .05; *n* ≥ 3). (D and E) MED6 and MED6-TWIST1 (2.6 × 10^4^ cells/well) cells were treated with vardenafil (10 µM) or vehicle (H_2_0) at days 0–4 in the 3D-BME model. (D) Time-lapse images were taken at ×10 magnification (scale bars represent 100 µm). (E) The mean aggregate area was quantified from time-lapse images taken between days 0 and 4 (unpaired *t*-test with Sidak–Bonferroni correction *P ≤ .05; *n* ≥ 2).

Since high TWIST1 expression was associated with patient metastatic disease and the invasive edge of medulloblastoma xenografts, we investigated the possibility that TWIST1 can increase cell aggregation due to its ability to promote cell migration. We therefore engineered a TWIST1 over-expressing cell line by lentiviral transduction of the non-metastatic MED6 and investigated the effect on cell aggregation. Expression analysis showed high levels of TWIST1 protein and qRT-PCR revealed an approximately 50-fold increase in TWIST1 gene expression in MED6-TWIST1 (ΔCt value ~0.2) compared to the MED6 parental cell line (ΔCt value see Supplementary Figure S3). The level of TWIST1 gene expression observed in MED6-TWIST1 was comparable with the MED1 cell line. In 3D culture, MED6-TWIST1 rapidly formed large cell aggregates (Figures 3I and 4D), which resembled the metastatic MED1 cell line (Figure 1B).

To further explore TWIST1’s potential in driving medulloblastoma cell migration, we inhibited its expression in the highly expressing TWIST1 metastatic cell lines D283Med (M2) and ONS-76 (Supplementary Figure S4A) either genetically using shRNA or chemically using Harmine.^36^ shRNA treatment resulted in a significant reduction in TWIST1 gene expression in the D283Med and ONS-76 cell lines by 63% and 68%, respectively, and a comparable reduction in protein expression (Supplementary Figure 4B and C). Treatment with Harmine (D283Med; 5 µM and ONS-76; 20 µM) caused a 77% and 70% reduction in TWIST1 protein expression, respectively.^36^ Subsequent to either chemical or genetic knockdown of TWIST1, a significant reduction in cell migration was observed without impacting cell viability (*P* ≤ .05; Figure 3J and K; Supplementary Figure S5).

### ABCB1 Inhibition Can Block Cell TWIST1-Driven Migration in Metastatic Medulloblastomas

We have previously correlated the expression of ABCB1 with high-risk medulloblastoma patients. Metastatic status is known for 259 of these patients enabling us to investigate a correlation between metastasis and ABCB1 expression.^20^ Positive ABCB1 expression was more likely to be observed in metastatic tumors (50.5%) compared to non-metastatic tumors (37.7%; *P* = .04; Figure 4A).

We have previously shown that the ABCB1 gene is expressed in medulloblastoma cell lines,^20^ and the overexpressing MED6-TWIST1 cell line showed a 4-fold increase in ABCB1 gene expression relative to parental MED6 cell line (Supplementary Figure S3B). We therefore set out to determine if TWIST1 could bind to the E-box located on the promoter region of *ABCB1* using a ChIP assay. TWIST1 is significantly bound to the E-box binding site in the Exon3 active promoter region of *ABCB1* (*P* = .03) and *GAPDH* (*P* = .018) in D283Med cells (Figure 4B and C; Supplementary Figure S6). However, TWIST1 did not bind to *SNAI2*, correlating to the previous results whereby *SNAI2* was not expressed in both 2D and 3D metastatic cell lines (Supplementary Figure S1).

We then analyzed whether inhibition of ABCB1 could affect cell aggregation in medulloblastoma cell lines cultured in the 3D-BME model. We treated the ABCB1-high MED1 and intermediate-expressing D283Med cell lines with the ABCB1-specific inhibitor, vardenafil, at a clinically relevant, nontoxic concentration^20,37^ (10 µM; Supplementary Figure S7A). Cell aggregation was significantly attenuated in both MED1 and D283Med (Supplementary Figure S7B and C) after exposure to vardenafil for 3 (MED1 *P* ≤ .01, D283Med *P* ≤ .05) and 4 days (MED1 *P* ≤ .05, D283Med *P* ≤ .05). This effect appeared to be more marked in the MED1 cell line, which also expresses high levels of TWIST1 (Supplementary Figure S1). Furthermore, the increased aggregation observed in the TWIST1-overexpressing MED6 cells could be significantly blocked by inhibition of ABCB1 function using vardenafil (*P* ≤ .05; Figure 4D and E). The rate of cell aggregation was significantly increased in MED6-TWIST1 compared to the MED6 parental cell line day 4 of 3D culture confirming that TWIST1 expression induces cell aggregation (Figure 4E).

## Discussion

Patients with metastatic disease respond poorly to therapy and are classified into high-risk categories.^38^ In this study, we investigated mechanisms of medulloblastoma metastasis using a patient-relevant culture system. The 3D-BME model incorporates major ECM components including laminin, collagen IV, entactin, heparan sulfate proteoglycans, and several growth factors to mimic the patient’s tumor microenvironment. In the 3D-BME model, non-tumorigenic/non-metastatic and metastatic cell lines displayed different growth patterns. All cell lines with the exception of immortalized cerebellar granule progenitor cells (C17.2; which remained as single cells) formed cell aggregates that displayed 2 out of 4 previously defined phenotypes (benign round, mass, grape-like, and invasive stellate). Neural stem cells, non-metastatic MED6, and UW228-3 as well as metastatic MED1 displayed a “stellate” morphology, a phenotype associated with invasiveness.^39^ Neural stem cells and non-metastatic cell lines however formed small cell aggregates which failed to sustain their metabolic activity, while the metastatic MED1 formed large cell aggregates which maintained their metabolic activity by continually migrating and combining. The D283Med and D458Med cell lines, derived from metastatic sites, displayed a “grape-like” morphology with generally smaller aggregates probably due to their weak cell–cell interactions and cell–matrix adhesions. These are properties that could facilitate the transit of disseminating tumor cells to metastatic sites as has been observed by others in aggressive breast^39^ and colorectal^40^ cell lines derived from metastases.

Live-cell imaging of the MED1 cell line showed directional movement between multiple cell clusters, a process known as chemotaxis-driven cell migration.^30^ The cell aggregation observed in our study has been recapitulated by Puliafito et al.^30^ where metastatic prostate cancer cells (PC3) cultured in BME also increased their aggregate size in a directional and chemotaxis-driven process. This phenotype is relevant during medulloblastoma dissemination in the patient, where reports provide evidence that secretory cytokines and growth factors could be responsible for promoting chemotaxis cell migration at the leptomeningeal membrane.^4,6^

To assess the relevance of our model, we assessed the expression of WIP1, a metastatic medulloblastoma marker, which promotes migration through crosstalk with the chemokine CXCR4 and G-protein-coupled receptor GRK5.^31,41^ WIP1 was significantly upregulated in the metastatic medulloblastoma cell line (M2; D283Med) compared to non-metastatic (M0; MED6 and UW228-3) and early-stage metastatic (M1; D458Med) cell lines. Buss et al. and others have demonstrated that CCT007093 inhibits the proliferation of WIP1-expressing cells.^32,41,42^ In 2D culture, WIP1 inhibition with 5 µM CCT007093 caused cell cytotoxicity. However, we show in 3D-BME, that 5 µM CCT007093 did not induce cytotoxicity but attenuated cell aggregation, hence demonstrating that in a relevant 3D model WIP1 inhibition has the expected effect on cell aggregation, thus validating the 3D-BME model.^31^

Emerging evidence identifying the existence of medulloblastoma circulating tumor cells during hematogenous dissemination has recently highlighted alternative routes of metastasis that have previously been overlooked. To this end, we assessed the role of the EMT transcription factor, TWIST1 in medulloblastoma cell migration. TWIST1 was exclusively expressed in aggregating cell lines in an in vitro model of medulloblastoma metastasis and at the migratory edge of an in vivo metastatic medulloblastoma model. TWIST1 was also associated with both metastasis at diagnosis and relapse in medulloblastoma patients. In the 3D-BME model, TWIST1 overexpression in MED6 cells (which previously failed to form metabolically active cell aggregates) induced cell aggregates that collectively migrated over 5 days. We knocked down TWIST1 using shRNA and degraded TWIST1 protein chemically using Harmine,^36^ allowing for further investigation of the functional importance of TWIST1 in medulloblastoma cell migration. Reduced TWIST1 expression in the D283Med and ONS-76 cell lines significantly decreased cell migration in the 3D transwell assay following either genetic or chemical knockdown of TWIST1. Both Harmine and shRNA knockdown of TWIST1 may have distinct off-target effects; however, the fact that both approaches inhibit migration confirms a specific role for TWIST1. This supports our hypothesis that, in common with several other types of cancer,^43^ TWIST1 is a master regulator of tumor metastasis in medulloblastoma.

TWIST1 has also been implicated in promoting chemoresistance and stemness by co-expression with ABC transporters.^44–46^ We have previously demonstrated that the multidrug-transporter ABCB1 contributes to chemoresistance in ABCB1-expressing medulloblastoma cell lines and that high expression is associated with high-risk medulloblastoma tumors.^20,22^ Here, we confirmed that ABCB1 is associated with metastasis in patients, providing further evidence linking ABCB1 with invasion.^47–49^ In support of this, we show that the clinically approved ABCB1 inhibitor vardenafil could be used to inhibit cell aggregation in ABCB1-expressing cell lines cultured in the 3D-BME models, with a more dramatic effect observed in MED6-TWIST1.^49,50^ Using ChIP pulldown analyses we also demonstrated that TWIST1 binds to the E-box on the promoter region of *ABCB1*. Further genetic knockdown experiments are required to elucidate how TWIST1 is regulating *ABCB1* and whether dual TWIST1/ABCB1 inhibition has the potential to prevent medulloblastoma metastasis.

In conclusion, we identified a role for TWIST1 during medulloblastoma metastasis and demonstrate the potential of therapeutically inhibiting ABCB1 with the FDA-approved vardenafil to prevent metastasis.

## Supplementary Material

vdab030_suppl_Supplementary_Materials

## Notes

**Authorship Statement** I.D.K., A.M.G., and B.C. led the study. A.N. and A.C. planned, executed the main body of in vitro experiments, ex-vivo, and patient analysis. R.O. assessed ABCB1 patient data and M.D., A.K., and S.M.P. provided German DKFZ patient arrays and associated data. N.N. generated the TWIST1 expression vector. D.O. provided technical expertise on the 3D-BME model. A.R. generated the in vivo model. A.L., H.C., C.V., and A.N. identified candidate markers. A.C. and F.L. planned and executed ChIP assays for ABCB1 and TWIST1. A.N., A.C., A.M.G., I.D.K., and B.C. wrote the manuscript. All authors were involved in editing drafts and approved the final version of the manuscript.
