# Corrigendum to: ABCB1 inhibition provides a novel therapeutic target to block TWIST1-induced migration in medulloblastoma

**DOI:** 10.1093/noajnl/vdab155

**Published:** 2021-12-23

**Authors:** Aishah Nasir, Alice Cardall, Ramadhan T Othman, Niovi Nicolaou, Anbarasu Lourdusamy, Franziska Linke, David Onion, Marina Ryzhova, Hanna Cameron, Cara Valente, Alison Ritchie, Andrey Korshunov, Stefan M Pfister, Anna M Grabowska, Ian D Kerr, Beth Coyle

**Affiliations:** 1 Children’s Brain Tumour Research Centre, Division of Child Health, Obstetrics and Gynaecology, School of Medicine, University of Nottingham, Nottingham, UK; 7 College of Medicine, University of Duhok, Kurdistan, Iraq; 2 Division of Cancer and Stem Cells, School of Medicine, University of Nottingham, Nottingham, UK; 3 School of Life Sciences, University of Nottingham, Nottingham, UK; 4 Department of Neuropathology, NN Burdenko Neurosurgical Institute, Moscow, Russia; 5 Cooperation Unit Neuro-oncology, German Cancer Research Center (DKFZ), Heidelberg, Germany; 6 Hopp Children’s Cancer Center Heidelberg (KiTZ), German Cancer Research Center (DKFZ), Division of Pediatric Neurooncology and Heidelberg University Hospital, Department of Pediatric Hematology and Oncology, Heidelberg, Germany

In the originally published version of this manuscript, the affiliation was incorrect for Ramadhan T Othman. The correct affiliation is College of Medicine, University of Duhok, Kurdistan, Iraq. This error has been corrected online.

