# Supplementary material for: ABCB1 inhibition provides a novel therapeutic target to block TWIST1-induced migration in medulloblastoma
Source: Neurooncol Adv. 2021 Apr 28;3(1):vdab030. doi: 10.1093/noajnl/vdab030 (PMC8080134; doi:10.1093/noajnl/vdab030)
Supplement: vdab030_suppl_Supplementary_Materials [file vdab030_suppl_supplementary_materials.zip › Supplementary Methods, Figures Videos and tables.docx]

**Supplementary Methods**

**Database search**

Analysis of RNA-Seq data of TWIST1 and SNAI2 was completed using medulloblastoma tumour samples and normal cerebellum tissue samples from the Pfister and Roth patient datasets, respectively, available on the R2: Genomics Analysis and Visualisation Platform (r2.amc.nl).

**Murine metastatic medulloblastoma model**

The murine model was produced using MED1-Fluc cells generated by lentiviral transduction of MED1 cells with the in-house luciferase expression vector (pLVX-luc) based on the Lenti-XTM Lentiviral Expression System (Clontech) according to manufacturer’s instructions to allow visualisation in vivo. All surgical procedures were performed using standard aseptic techniques, with sterile gloves, instruments and drapes used throughout the procedure. The mice (MF-1 NuNu males, obtained from Harlan, UK, at 8-12 weeks of age), after a week’s acclimatisation, were anaesthetised with an injectable anaesthetic combination (Anaestemine [ketamine]/Sedastart [medetomadine], Animalcare Ltd. UK), and prepared by administering Rimadyl (Carprofen, Zoetis) and an opthalmic ointment (Lacri-lube, Allergan Inc.) prior to surgery. After prepping the surgical area with chlorhexidine (Hibiscrub, VetTech, UK), the mOuse was restrained in a stereotaxic frame (Stoelting, Harvard Apparatus, UK), a front to back incision was made on the animal’s head, the membrane was carefully scraped a at 7mm posterior to bregma, 1mm right of the midline, way from the skull with a 26G needle and a burr hole was created using a dental drill (Ram Products Microtorque II, Harvard Apparatus, UK) at 7mm posterior to bregma, 1mm right of the midline. MED1-Fluc cells with viability of >90%, which had been maintained in vitro in tumour media and selected with puromycin (4µg/ml) weekly, were injected at 6x104 cells in 5µl of PBS (12x106 cells/ml) per mouse, via the burr hole, at a depth of 3mm, using a 26-gauge Hamilton Gastight 1701 syringe needle, and the wound was sutured closed with Vicryl (W9074, Ethicon, UK), followed by appropriate post procedural monitoring and therapy, including placing the mice on a heat pad and providing fluid replacement via wet mash once awake. They were further treated with another dose of Rimadyl 24 hrs post-surgery.

Tumour growth was monitored with bioluminescence whole-body imaging of mice, performed twice weekly upon D-Luciferin (120 mg/kg by weight) injection using the IVIS Spectrum (Caliper Life Sciences). Prior to imaging, the mice were again anaesthetised as above, before being placed in the imaging system and, post imaging, being allowed to recover from the anaesthetic as above.

Mice were maintained in Individually Ventilated Cages (Tecniplast UK) within a barriered unit, illuminated by fluorescent lights set to give a 12 hour light-dark cycle (on 07.00, off 19.00), as recommended in the guidelines to the Home Office Animals (Scientific Procedures) Act 1986 (UK). The room was air-conditioned by a system designed to maintain an air temperature range of 21 ± 2ºC and a humidity of 55% + 10%. Mice were housed in social groups, 3 per cage, during the study, with bedding, nesting materials and environmental enrichment (Datesand UK). Sterile irradiated 5V5R rodent diet (IPS Ltd, UK) and autoclaved water was offered *ad libitum.* The condition of the animals was monitored throughout the study by an experienced animal technician. All animal experiments were performed in accordance with the United Kingdom Animals (Scientific Procedures) Act 1986, under the UK Home Office project license authority PPL40/3559. Ethical approval was granted by the University of Nottingham Animal Welfare and Ethical Review Board.

**Cell Viability assay**

Cell viability of Harmine treated cells was measured using the Trypan Blue assay (Fisher, 1:2 dilution).

**Western Blot analysis**

Protein from total cell lysates were resolved on sodium dodecyl sulfate polyacrylamide (SDS-PAGE) gels and transferred onto PVDF membranes (GE Healthcare) for immunoblotting. Blots were probed with mouse anti-TWIST1 (anti-Twist2Clα mouse monoclonal Ab; Abcam 1:100), and mouse anti-β-actin (Sigma-Aldrich 1:1000) as a loading control. Primary antibodies were detected using anti mouse horseradish peroxidase-conjugated antibodies (1:2500) and enhanced chemoluminescence (SignalFire ECL Reagent, Cell Signaling Technology) was performed according to the manufacturer’s protocol.

**Supplementary tables**

**Table S1: Clinicopathological characteristic of MB patients included in the Birmingham TMA in Figure S2 and 3.**

| **Variable** | **No** | **%** |
| --- | --- | --- |
| **Gender** |  |  |
| F | 9 | 21 |
| M | 33 | 79 |
| **Age, years** |  |  |
| <3 | 8 | 19 |
| ≥3 | 34 | 81 |
| **Metastatic status** |  |  |
| M- | 24 | 57 |
| M+ | 18 | 43 |
| **Resection status** |  |  |
| Complete | 26 | 62 |
| Incomplete | 16 | 38 |
| **Histology** |  |  |
| Classical | 17 | 40 |
| Desmoplastic | 6 | 14 |
| LC/A | 5 | 12 |
| unknown | 14 | 33 |
| **Recurrence** |  |  |
| Yes | 19 | 45 |
| No | 23 | 55 |
| **Subgroup** |  |  |
| WNT | 4 | 10 |
| SHH | 6 | 14 |
| Group 3 | 7 | 17 |
| Group 4 | 14 | 33 |
| Unknown | 11 | 26 |
| **Status** |  |  |
| A | 20 | 48 |
| D | 21 | 50 |
| Unknown | 1 | 2 |
| Abbreviations: M= male, F= female, LC/A= large cell/anaplastic, A= alive, D= dead. | | |

**Table S2: Antibodies used for IHC analysis used in Figure 3 and 4.**

| **Primary Ab** | **Supplier** | **Species** | **Dilution** | **Antigen Retrieval** | **Incubation** | **Staining pattern** | **Positive control** |
| --- | --- | --- | --- | --- | --- | --- | --- |
| **TWIST1**  **(ABD29)** | Millipore | Mouse | 1:250/  1:500 | Steamer | 1 hour at RT | Nuclear | Tonsil |
| **ABCB1**  **(C219)** | Calbiochem | Rabbit | 1:40 | Pressure cooker | Overnight at 4^o^C | membranous | Liver |

**Table S3: Seeding densities for cell lines cultured in the 3D-BME model in Figures 1, S1, 2, 3, S3, 4 and S6.**

| **Cell line** | **Seeding densities**  **cells/well** |
| --- | --- |
| D283 Med | 3.5x10^4^ |
| D458 Med | 6.0x10^4^ |
| MED1 | 1.5x10^4^ |
| MED6 | 2.5x10^4^*/ 3.5x10^4^ |
| MED6-TWIST1 | 2.5x10^4^* |
| UW228-3 | 1.5x10^4^ |
| FB83 | 2.5x10^4^ |
| C17.2 | 2.5x10^4^ |
| C17.2-Wnt1 | 2.5x10^4^ |
| MCF-7vsvgR2 | 1.25x10^4^ |
| MDA-MB-231Re4XAA | 1.25x10^4^ |
| * The optimal seeding density for MED6 parental was 3.5x10^4^ cells/well (used for growth assays; see fig1). Drug treatment assays were carried at 2.5x10^4^ cells/well to account for the MED6-TWIST1 slow growth rate (see fig4). | |

**Table S4: Sequence of primers used for qRT-PCR analysis in Figure S1, 2, 3 and S3**

| **Gene** | **Forward Primer** | **Reverse Primer** |
| --- | --- | --- |
| **ABCB1** | 5’ CCCATCATTGCAATAGCAGG 3’ | 5’ GTTCAAACTTCTGCTCCTGA 3’ |
| **GAPDH** | 5’ ATGTTCGTCATGGGTGTGAA 3’ | 5’ GTCTTCTGGGTGGCAGTGAT 3’ |
| **SNAI2** | 5’ CTGCGGCAAGGCGTT 3’ | 5’ GCAGTGAGGGCAAGAAAAAGG 3’ |
| **TWIST1** | 5’ CAAGCTGAGCAAGATTCAGACCC 3’ | 5’ AGACCGAGAAGGCGTAGCTGA 3’ |
| **WIP1** | 5' AGGCTTTCTCGCTTGTCACC 3' | 5' TGTGCTAGGAAGACCCGTCA 3' |

**Table S5: Sequence of primers used for ChIP qRT-PCR analysis in Figure 4 and S5**

| **Gene** | **Forward Primer** | **Reverse Primer** |
| --- | --- | --- |
| **ABCB1** | 5’ GTCATCTGTGGTGAGGCTGA 3’ | 5’ AGGCTTCCTGTGGCAAAGAG 3’ |
| **GAPDH** | 5’ TCTTTGCAGTCGTATGGGGG 3’ | 5’ GGGAGTAGGGACCTCCTGTT 3’ |
| **SNAI2** | 5’ CAAACCTCTCCAGATGCCACT 3’ | 5’ TGTCCGCCAGGAGAAGGAAG 3’ |

**Table S6: Kaplan Meier analysis for predictors of overall survival and event free survival in medulloblastoma patient TMA’s**

| **Clinical  parameter** | **Overall Survival  (*p* value)** | **Event Free Survival  (*p* value)** |
| --- | --- | --- |
| Metastatic status | 0.000 | 0.017 |
| Age (3 years) | 0.111 | 0.181 |
| Extent of surgery | 0.140 | 0.716 |
| Gender | 0.483 | 0.328 |
| Molecular subgroup | 0.036 | 0.007 |

**Supplementary figures**

**Supplementary Figure S1**


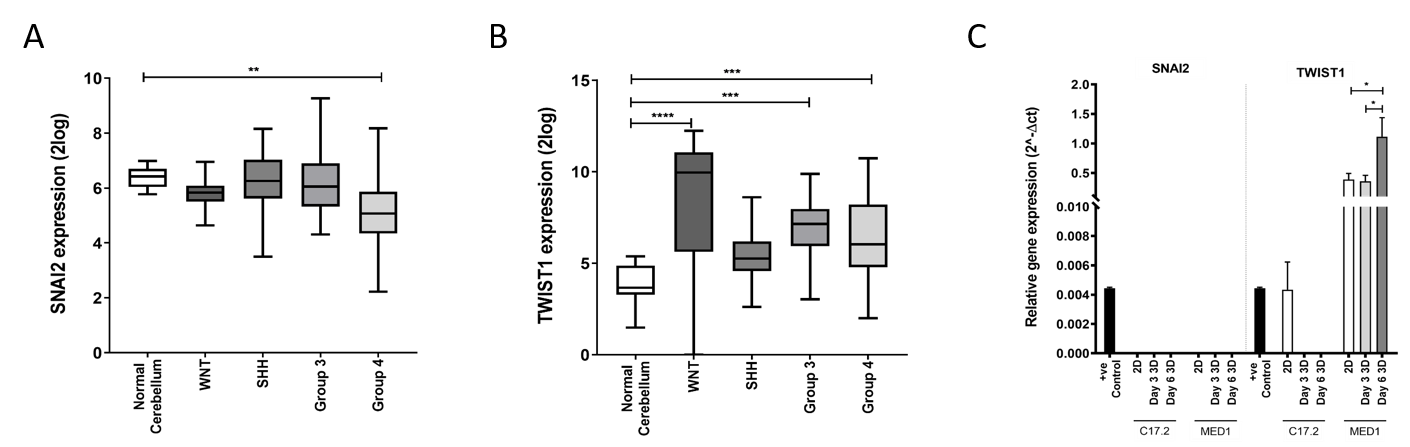


**Supplementary Figure S1: Expression analysis of EMT genes.** **A-B** Gene expression of the EMT factors SNAI2 and TWIST1 in 223 medulloblastoma patient samples and 9 normal tissues samples using the Pfister and Roth datasets, respectively, from the R2 database. Statistically significant results were calculated using one-way ANOVA with Dunnett’s multiple comparison post-hoc test comparing to normal cerebellum *(**p<0.01, ***p<0.001, ****p<0.*0001). **C** Relative gene expression of EMT factors SNAI2 and TWIST1in 2D and 3D samples of C17.2 and MED1 cell lines calculated against GAPDH using the 2-^ΔCt^ method. Statistically significant results were calculated by performing one-way ANOVA with Tukey’s multiple comparison post-hoc test (* *p*≤0.05) (n≥2).

**Supplementary Figure S2**


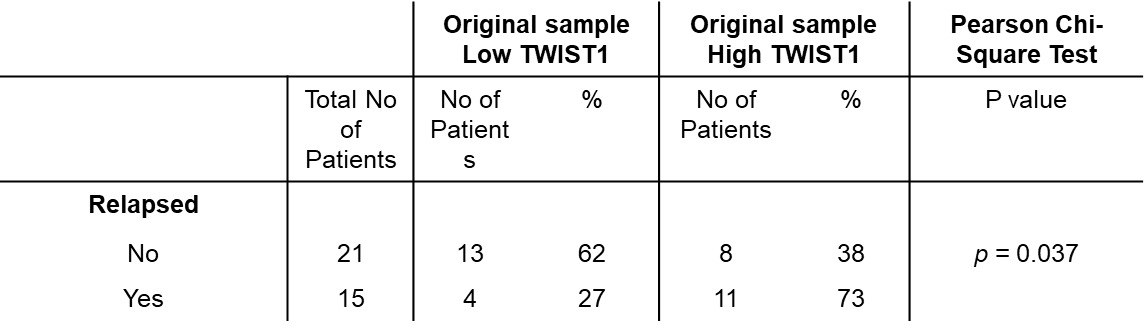


**Supplementary Figure S2: TWIST1 expression in patient TMA’s**. Follow up data on patients categorised as M0 from the nuclear TWIST1 IHC staining of Nottingham and Birmingham TMA’s. Statistical significance was calculated using a Pearson’s Chi-Squared test (*p*=0.037)

**
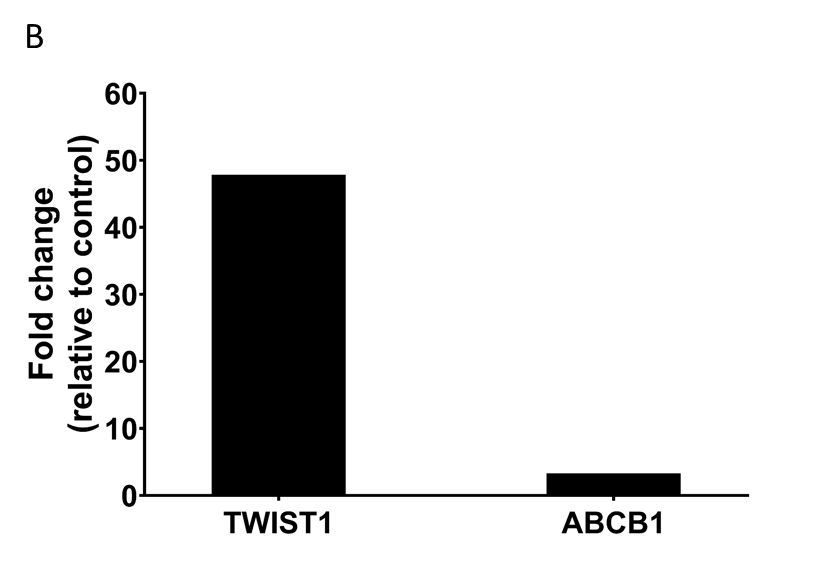
Supplementary Figure S3**

**
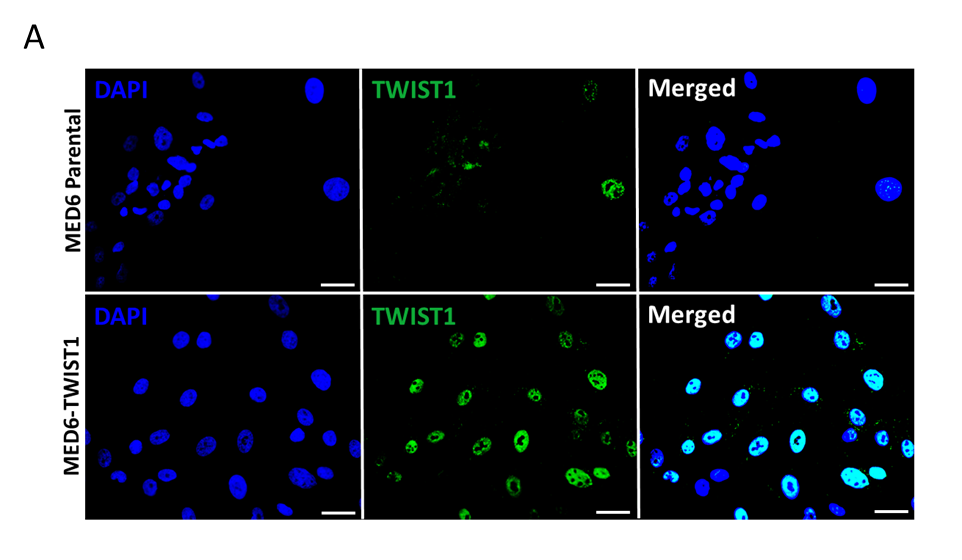
Supplementary Figure S3:** **Overexpression of TWIST1 in the MED6 cell line.
A** Immunofluorescence staining showing TWIST1 expression (green) in MED6 parental and MED6-TWIST1 cell lines. Nuclei were visualised with DAPI staining (blue). Merged DAPI and TWIST1 images are also shown. Images were taken at x20 magnification (scale bars represent 100µm). **B** Relative gene expression of ABCB1 and TWIST1 in 2D samples of MED6-TWIST1 (n=1) and MED6 parental cell line (n≥2) was calculated against GAPDH using the 2-ΔCt method. Fold change increase of TWIST1 and ABCB1 in MED6-TWIST1 was calculated relative to the MED6 parental cell line.

**
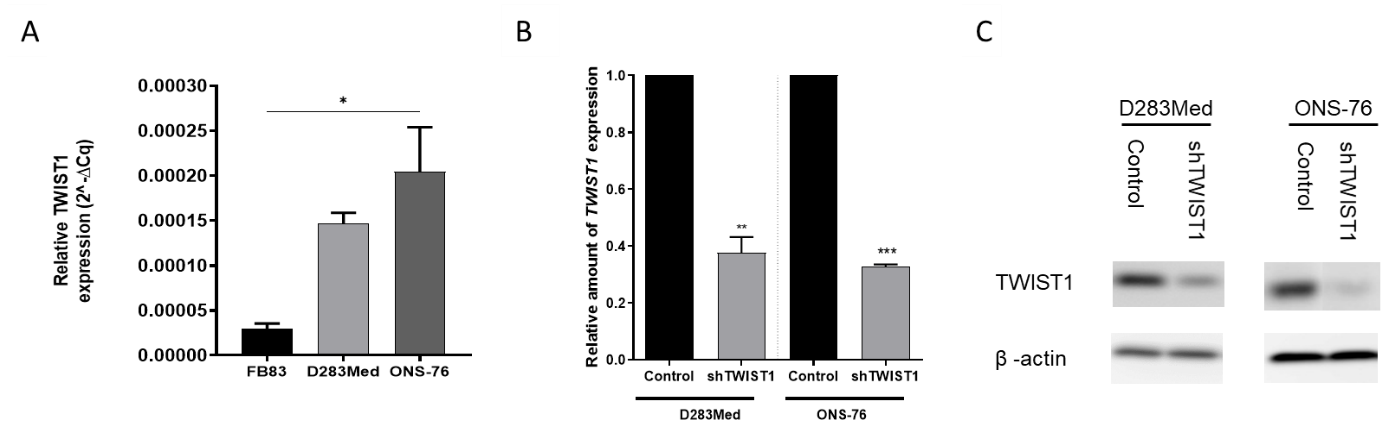
Supplementary Figure S4**

**Supplementary Figure S4: shRNA mediated knockdown of TWIST1 expression in D283 and ONS-76 cell lines.**

**A** Relative gene expression of TWIST1 in FB83, D283Med and ONS-76 cells lines calculated against GAPDH using the 2-^ΔCt^ method. Significant differences in gene expression compared to the FB83 cell lines were calculated by performing a one-way ANOVA with Dunnett’s multiple comparison post-hoc test (** p*≤0.05) (n=3). **B** Relative gene expression levels of TWIST1 in D283Med and ONS-76 cell lines after shRNA treatment compared to the non-targeting control. Significant differences were calculated using a paired Student’s t-test (***p* ≤ 0.01, ****p* ≤ 0.001 n=3). **C** Protein expression levels of TWIST1, after knocking down by shRNA, were detected by western blot analysis. β-actin served as a loading control. **Supplementary Figure S5**

**
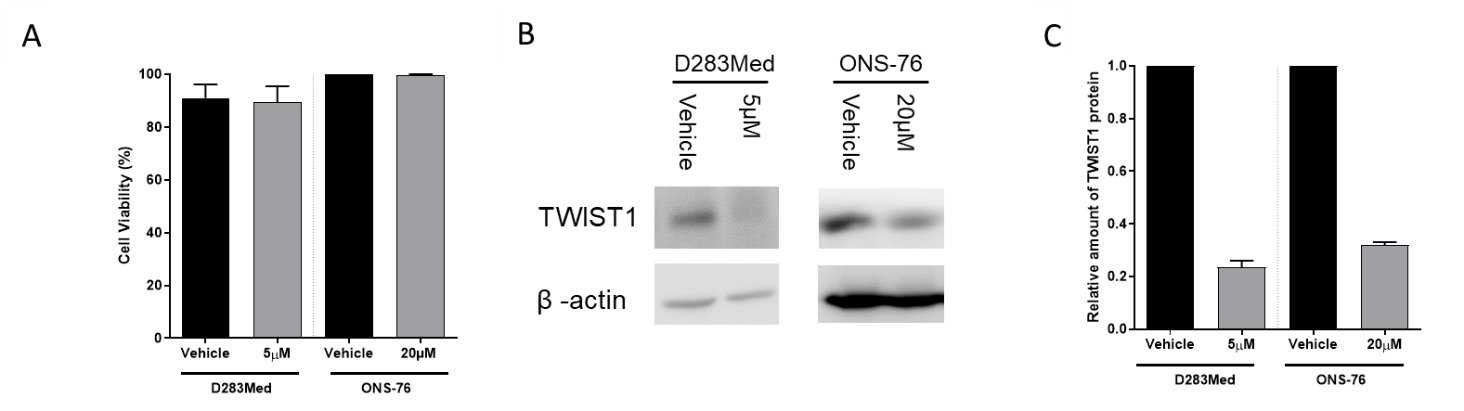
**

**Supplementary Figure S5: D283 and ONS-76 cell viability and TWIST1 protein expression after Harmine treatment.
A** Cell viability was assessed using trypan blue after D283Med and ONS-76 cells were treated with Harmine (5µM and 20µM, respectively) for 72 hours (n≥2) **B** Protein expression levels of TWIST1, after treatment with Harmine for 72 hours, were detected by western blot analysis. β-actin served as a loading control. **C** Protein expression was quantified using Fiji imaging software (n=2).

**Supplementary Figure S6**


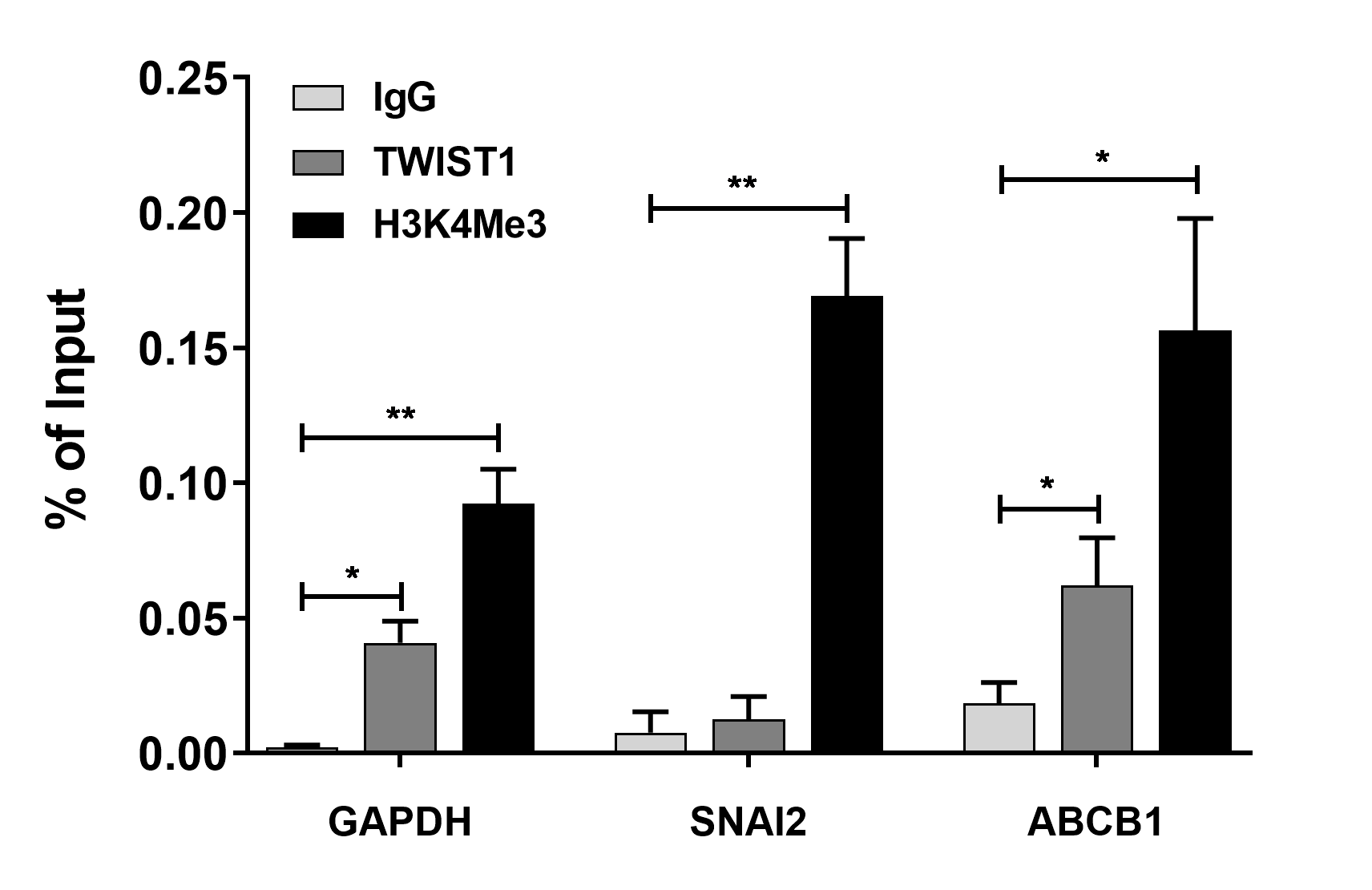


**Supplementary Figure S6: ChIP analysis of TWIST1 binding to active gene promoters.** ChIP qPCR analysis of TWIST1 binding to functionally active promoter regions of *GAPDH, SNAI2* and *ABCB1*. Data is calculated and expressed as a % of the input (n≥2). Significance was calculated using a paired Student’s t-test, comparing either the TWIST1 or H3K4Me3 IP’s against the negative control (IgG1). (** p* ≤ 0.05, *** p* ≤ 0.01).

**
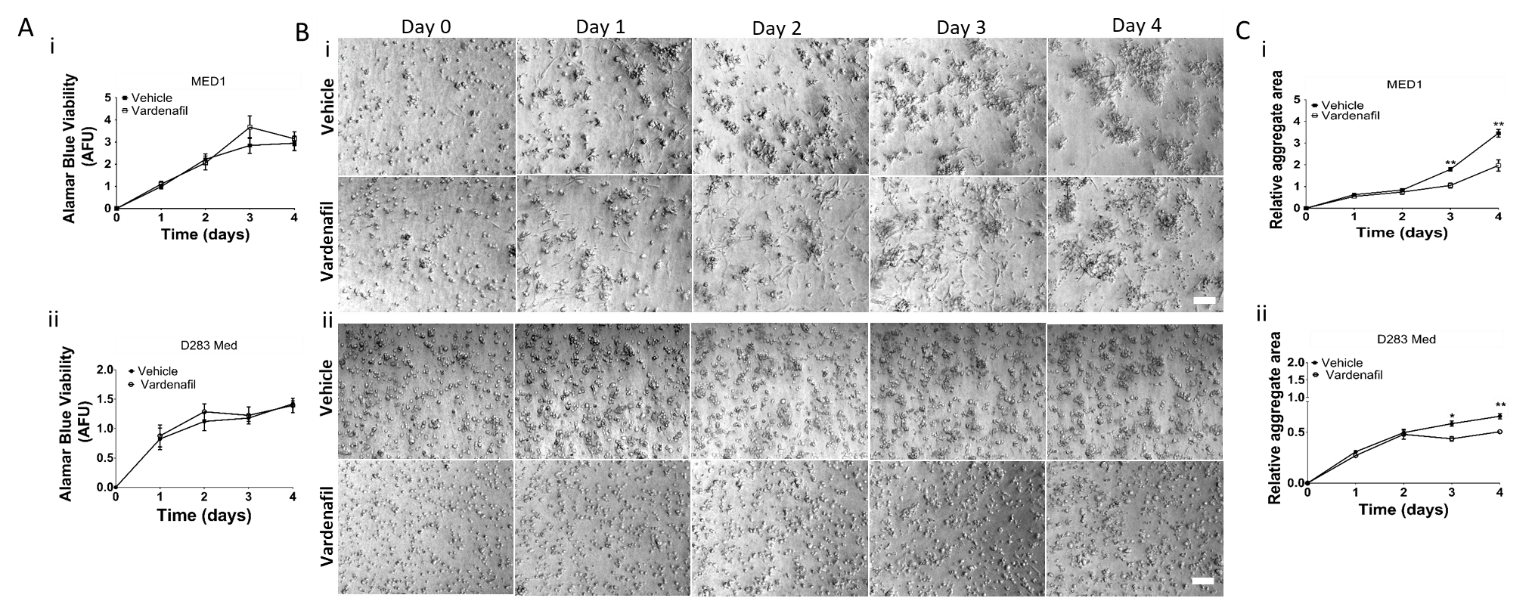
Supplementary Figure S7**

**Supplementary Figure S7: Inhibition of ABCB1 in the MED1 and D283 cell line A-C** MED1 (i) (1.5x10^4^cells/well) and D283Med (ii) (3.5x10^4^ cells/well) were vardenafil (10µM) or vehicle (H_2_0) treated at days 0-4 in the 3D-BME model. **A** Metabolic activity was assessed with the alamar blue assay and **B** time-lapse images were taken at x10 magnification (scale bars represent 100µm). **C** The mean aggregate area of MED1 and D283Med was quantified for each condition from time-lapse images (unpaired t-test with Sidak-Bonferroni correction ** p≤0.05* and *** p≤0.01*; n=3).

**Supplementary videos**

**Supplementary Video S1**

**Supplementary Video S1**: Live-cell imaging (Cell-IQ) of MED1 in the 3D-BME model during days 1-3 of culture.

**Supplementary Video S2**

**Supplementary Video S2:** Live cell imaging (Cell-IQ) of MED1 in the 3D-BME model during day 3-6 of culture.
